# Application of a Dy_3_Co_0.6_Cu_0.4_H*_x_* Addition for Controlling the Microstructure and Magnetic Properties of Sintered Nd-Fe-B Magnets

**DOI:** 10.3390/ma12244235

**Published:** 2019-12-17

**Authors:** Katerina Skotnicova, Pavel A. Prokofev, Natalia B. Kolchugina, Gennady S. Burkhanov, Alexander A. Lukin, Yurii S. Koshkid’ko, Tomas Cegan, Henryk Drulis, Tatyana Romanova, Nikolay A. Dormidontov

**Affiliations:** 1 VSB–Technical University of Ostrava, Faculty of Materials Science and Technology, 70800 Ostrava, Czech Republic; tomas.cegan@vsb.cz; 2 Joint Stock Company «Spetsmagnit», Moscow 127238, Russia; pav3387@yandex.ru (P.A.P.); lukinaalukin@rambler.ru (A.A.L.); 3 Baikov Institute of Metallurgy and Materials Science, Russian Academy of Sciences, Moscow 119334, Russia; natalik014@yandex.ru (N.B.K.); genburkh@imet.ac.ru (G.S.B.); yurec@mail.ru (Y.S.K.); ontip@mail.ru (N.A.D.); 4 Institute of Low Temperature and Structure Research, Polish Academy of Sciences, 50–422 Wroclaw, Poland; HDrulis@int.pan.pl (H.D.); T.Romanova@int.pan.wroc.pl (T.R.)

**Keywords:** grain boundary diffusion, Nd–Fe–B magnets, hydrogenation, microstructure, magnetic properties

## Abstract

The focus of new technologies on the formation of inhomogeneous distributions of heavy rare-earth metals (REMs) in hard magnetic Nd–Fe–B materials is of scientific importance to increase their functional properties, along with preserving existing sources of heavy REMs. This paper focused on the coercivity enhancement of Nd_2_Fe_14_B-based magnets by optimizing the microstructure, which includes the processes of grain boundary structuring via the application of a Dy_3_Co_0.6_Cu_0.4_H*_x_* alloy added to the initial Nd–Fe–B-based powder mixtures in the course of their mechanical activation. We have studied the role of alloying elements in the formation of phase composition, microstructure, the fine structure of grains, and the hysteretic properties of hard magnetic Nd(R)_2_Fe_14_B-based materials. It was shown that the Dy introduction via the two-component blending process (the hydrogenated Dy_3_Co_0.6_Cu_0.4_ compound is added to a powder mixture) resulted in the formation of the core-shell structure of 2–14–1 phase grains. The efficient improvement of the coercivity of Nd(RE)–Fe–B magnets, with a slight sacrifice of remanence, was demonstrated.

## 1. Introduction

Researchers have made many attempts to reduce the heavy rare-earth (RE) consumption Nd–Fe–B sintered magnets with high-coercivity. Some progress has been achieved using Dy and/or Tb in various forms to realize approaches named grain boundary diffusion (GBD) [1,2,3] and grain boundary structuring (GBS) [4,5,6,7,8]. The application of binary mixtures allows one to improve the structure of the boundary phases and grain boundaries of the main magnetic phase and to realize the diffusion of a required component of the alloy directly through the boundaries. It has been demonstrated that by controlling the process time and temperature of GBD processes, the coercivity of the magnet can be greatly enhanced, without sacrificing the remanence.

It was shown in our previous studies that hydrogenated Tb and Dy additions allowed us to enhance the coercivity with a slight decrease in the remanence [9] and increase the stability of the magnet properties during annealing at the low-temperature [10], respectively.

The grain boundary restructuring, with rare-earth-rich low-melting compounds added to low-alloyed Nd–Fe–B-based compositions in the course of technological processing, was realized when using (Pr,Nd)_6_Fe_13_Cu [4], Dy_32.5_Fe_62_Cu_5.5_ [5], Dy_69_Ni_31_ [6], Dy_88_Mn_12_ (wt.%) [11], Pr_34.4_Co_65.6_ (wt.%) [12], and Dy_82.3_Co_17.7_ (wt.%) [13], which is a low-melting eutectic composition. It was shown that the intrinsic coercivity evidently increased when using Dy_82.3_Co_17.7_ and the maximum intrinsic coercivity was achieved when its content was 2 wt.%. At the same time, the remanence and maximum-energy product decreased slightly as the Dy_82.3_Co_17.7_ content increased. By adding a small amount of Dy_82.3_Co_17.7_, the coercivity improved greatly, and the irreversible loss decreased sharply. The increase in the Curie temperature of the magnets suggests that Co atoms have been incorporated into the 2:14:1 main phase. A well-developed a core–shell structure is formed in these magnets.

The experiments with REM-M-H compounds (rare earth metal-transition metal(s)-hydrogen), which are added at the stage of mechanical milling and alloying, were performed to realize the optimum microstructure, nano-heterogeneous distribution of heavy REMs (Dy or Tb) within a grain, and economically alloyed composition of magnets, which assumes, in particular, the distribution of heavy REMs within the near-grain boundary areas. Such a heavy-REM distribution allows us to (1) locally increase the coercive force and decrease the probability of the formation of reverse domains at grain boundaries; (2) limit the substitution of heavy REM for neodymium in the matrix phase and, thus, decrease the probability of decreasing magnetization and remanence; and (3) decrease the amount of heavy REMs, which is required to reach the given increase in the coercive force. The latter circumstance determines the possibility of the development of physico-chemical and technological foundations of resource-saving technology, the possibility of decreasing the material costs and prices of products manufactured from the new alloys, and the possibility of substantially widening the functionality of the materials.

Thus, by applying compositions with a heavy rare-earth metal, the outer region of the Nd_2_Fe_14_B matrix grains was enriched during the sintering process and substitutes for Nd were used in the matrix grains to form the (Nd,Dy)_2_Fe_14_B core–shell phase. 

This paper focused on optimizing the microstructure of the near-stoichiometric Nd_2_Fe_14_B-based magnet, which included the grain boundary diffusion and grain boundary structuring processes via the application of a hydrogenated Dy_3_Co_0.6_Cu_0.4_H*_x_* composition added to a powder mixture.

## 2. Experimental

The strip casting technique was used for the preparation of the base Nd-24.0, Pr-6.5, Dy-0.5, B-1.0, Al-0.2, Fe-balance alloy (wt.%). The strip-cast alloy was subsequently subjected to hydrogen decrepitation process, which was realized during heating to 270 °C in a hydrogen flow at a pressure of 0.1 MPa and holding at this temperature for 1 h.

The Dy_3_(Co_1−x_Cu*_x_*) alloy with *x* = 0.4 was produced by the arc melting of the starting components (distilled Dy of 99.9% purity, Co of ≥ 99.25% purity, and oxygen-free Cu of 99.95% purity) in an argon atmosphere using a water-cooled copper bottom and a non-consumable tungsten electrode. The ingot was homogenized at 600 °C for 90 h and subjected to hydrogenation under conditions used for the strip-casting alloy, namely, upon heating to 270 °C in a hydrogen flow at a pressure of 0.1 MPa and subsequent 1 h heating at this temperature (Regime 1 was used to manufacture the magnet), and upon heating to 700 °C in a high-purity hydrogen atmosphere and holding at this temperature for 1 h in a glass Sieverts-type apparatus (Regime 2 was used for investigations). In the case of heating at 700 °C, the hydrogenation up to the Dy_3_Co_0.6_Cu_0.4_H*_x_* composition with *x* = 8.26 was realized. It is expected that such a hydrogen content accords with the complete hydrogenation of dysprosium to a dysprosium hydride.

The mixture of hydrogen-decrepitated strip-cast Nd(RE)–Fe–B alloy and the Dy_3_Co_0.6_Cu_0.4_H*_x_* alloy (Regime 1) was milled for 40 min to an average particle size of 3 μm using a vibratory mill and isopropyl alcohol medium. After wet pressing of the pulp in a transverse magnetic field of 1500 kA/m, compacts were sintered at 1080 °C for 2 h and optimally heat treated (HT) at 500 °C for 2 h. Then, samples of the magnet were subjected to low-temperature heat treatment in the temperature range 400–900 °C, with subsequent quenching in N_2_. 

The phase composition of the Dy_3_Co_0.6_Cu_0.4_ and Dy_3_Co_0.6_Cu_0.4_H*_x_* (*x* = 8.26) alloys was investigated by X-ray diffraction (XRD) analysis using an Ultima IV (Rigaku») diffractometer (equipped with a “D/teX” detector, CuKα radiation) and a Philips X’Pert 1 diffractometer, respectively; the scanning step was 0.001°. X-ray diffraction patterns were processed, and the phase composition of the alloy was determined using PowderCell software. Data on the crystal structure type, lattice parameters, and the crystallographic positions of atoms in the Dy–Co, Dy–Cu, and H–Dy systems [14,15,16] were used to simulate theoretical XRD patterns. 

An Quanta 450 FEG high-resolution field emission gun scanning electron microscope (FEI Company, Fremont, USA) equipped with an energy-dispersive spectroscopy (EDS, EDAX Inc., Mahwah, USA) microprobe was used to investigate the structure, chemical composition, and distribution of magnet components (X-ray mapping) of the addition and magnet sample. The mean particle size was evaluated by means of a MasterSizer 3000 laser diffraction particle size analyzer (Malvern Panalytical Ltd, Malvern, United Kingdom). The hysteretic properties of the magnet sample were measured at room temperature (RT) using an automatic hysteresis graph system MH-50 (Walker Scientific Inc., Worcester, USA). The differential thermal analysis (DTA) and thermogravimetric analysis were performed under an argon atmosphere with a heating/cooling rate of 30 °C/min using a STA 449 F3 Jupiter installation (Netzsch Holding, Selb, Germany). 

## 3. Results and Discussion

### 3.1. X-Ray Diffraction Analysis

**Figure 1:**
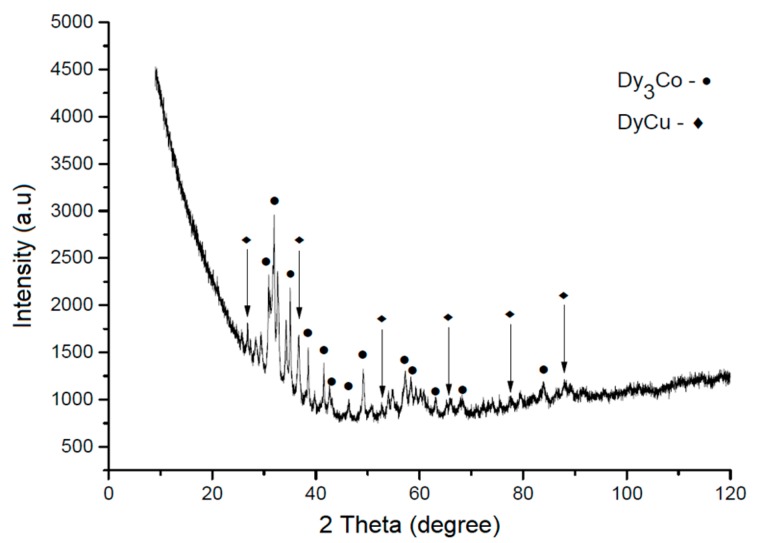
X-ray diffraction pattern of the Dy_3_Co_0.6_Cu_0.4_ alloy.

Figure 1 shows the X-ray diffraction pattern of the Dy_3_Co_0.6_Cu_0.4_ alloy subjected to prolonged annealing in an argon atmosphere. The reflections belong to the main Dy_3_(Co,Cu) phase and the Dy(Cu,Co) phase based on DyCu [14,15]. The analysis of the crystal structures of the found compounds and theoretical XRD patterns constructed for the simulated structures allowed us to determine variations in the lattice parameters of the Dy(Cu_1−y_Co_y_) and Dy_3_(Co_1−x_Cu_x_) phases alloyed with Co and Cu, respectively (see Table 1). As seen, the alloying of the binary compounds with Co and Cu did not change the crystal structure type of the compounds. In accordance with the binary phase diagrams [14,15], the phases present in the alloy are alloyed compositions of the binary compounds. 

**Figure 2:**
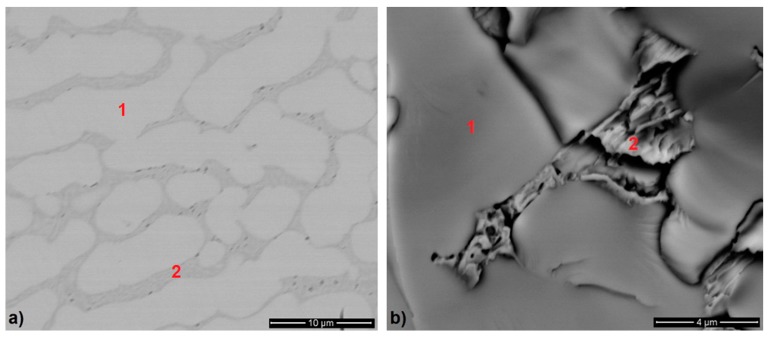
Scanning electron microscopy images of the microstructure of Dy_3_Co_0.6_Cu_0.4_ alloy subjected to prolonged annealing in an argon atmosphere: (**a**) metallographic section, and (**b**) fracture surface.

The phase composition of the alloy was also confirmed by the EDS microanalysis, see Figure 2 and Table 2. The microstructure consisted of Dy_3_(Co_1−x_Cu_x_) (*x* ~ 0.4) dendrites (point 1 in Figure 2) and Dy(Cu_1−y_Co_y_) + Dy_3_(Co_0.6_Cu_0.4_) mixture (point 2 in Figure 2) found in the interdendritic regions. The composition of the Dy(Cu_1−y_Co_y_) phase cannot be accurately determined by the EDS analysis because of its small size, since the surrounding matrix is analyzed along with this very small inclusion. However, the increased content of copper is evident in this mix area.

As is shown in Table 1, the substitution of Cu for Co in Dy_3_(Co_1−x_Cu_x_) (with regard to the solubility of Cu and Co in Dy_3_Co and DyCu, respectively) changed the lattice parameters: the lattice parameters *b* and *c* increased as the radius of Cu atoms (0.128 nm) was higher than that of the Co atoms (0.125 nm), whereas the lattice parameter *a* decreased. This is likely to be due to the fact that copper atoms substitute for cobalt atoms only at certain sites.

We assumed that the solidification of the alloy occurs via the primary formation of the Dy_3_Co-based phase by peritectic reaction; the DyCu-based compound is the secondary phase. According to the Co–Dy phase diagram, the solidification path may include the formation of the Dy_12_Co_7_-based phase by peritectic reaction.

### 3.2. Interaction of Dy_3_(Co_,_Cu) Alloy with Hydrogen

**Figure 3:**
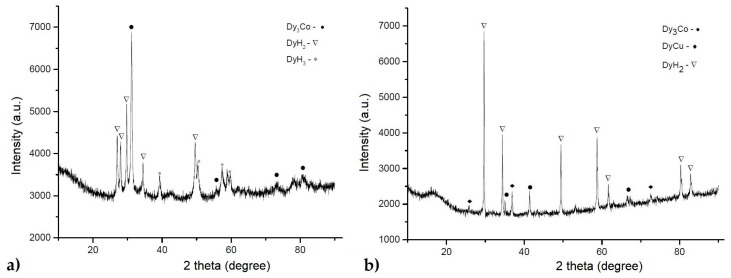
X-ray diffraction pattern of the Dy_3_(Co,Cu) alloy after saturation with hydrogen (Regime 2) (**a**) and after the thermal dehydrogenation process (DTA) (**b**).

The saturation of the Dy_3_Co_0.6_Cu_0.4_ alloy with hydrogen led to the embrittlement of the alloy (i.e., the powder material suitable for further introduction of the composition into the Nd–Fe–B magnetic alloy powder during cooperative milling was obtained). Figure 3a shows the X-ray diffraction analysis data for the Dy_3_Co_0.6_Cu_0.4_ alloy subjected to hydrogenation (Regime 2). The hydrogenated composition contained DyH_2_ [17] and DyH_3_ [18] hydrides. Other reflections corresponded to the Dy_3_(Co,Cu) phase; it is likely that small quantities of the Dy_3_(Co,Cu) and Dy(Cu,Co) phases did not react with hydrogen. After hydrogenation, copper and cobalt may be present in the form of a fine mixture.

Figure 3b shows the X-ray diffraction analysis data of the alloy Dy_3_Co_0.6_Cu_0.4_H*_x_* subjected to thermal dehydrogenation (upon heating during DTA). The sample was heated up to 700 °C (Figure 3). After heating, the presence of DyH_2_ and small quantities of the Dy_3_(Co,Cu) and Dy(Cu,Co) phases were detected; DyH_3_ was absent. The presence of a thin mechanical mixture of Cu and Co is also possible.

**Figure 4:**
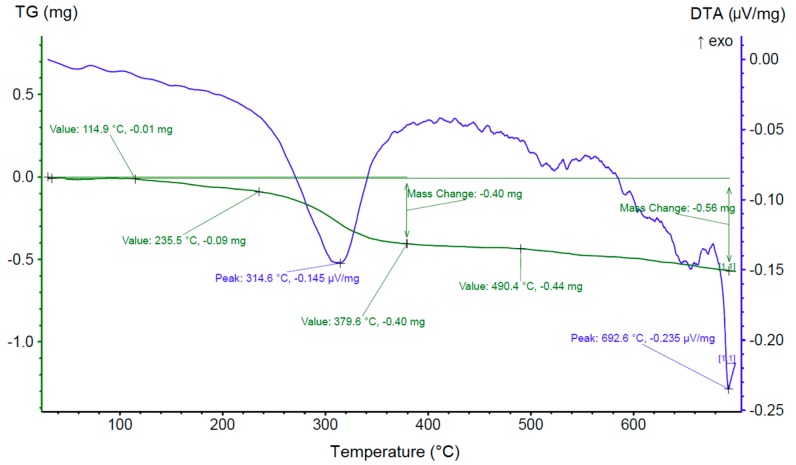
Thermogravimetric analysis (TG) and differential thermal analysis (DTA) curves of the Dy_3_(Co,Cu)H_8.26_ sample.

According to the DTA data (Figure 4), the decomposition of DyH_3_ started at a temperature of ~314 °C, which agreed with the literature data [16]. Between ~314 °C and ~690 °C, no thermal effects were identified. Above ~690 °C, in accordance with the Dy–H [16] diagram, the solid solution of hydrogen in dysprosium decomposed to form dysprosium. However, the thermal effects at temperatures above 600 °C can correspond to the melting of one of the metallic phases of the alloy; nevertheless, the thermal effect corresponding to ~690 °C is accompanied by a significant weight loss. The observed formation of Dy hydrides indicates the possibility of the hydrogenated Dy_3_Co_0.6_Cu_0.4_ alloy to be used as additions in manufacturing sintered Nd–Fe–B magnets.

### 3.3. Microstructure and Electron Microprobe Analysis of Sintered NdFeB-Based Magnet

**Figure 5:**
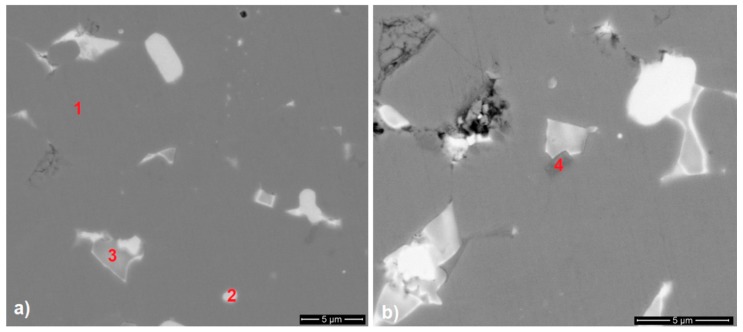
The microstructure of the Nd–Fe–B sintered magnet prepared from the powder mixture with 2 wt.% Dy_3_Co_0.6_Cu_0.4_H_x_; (**a**) phase 1—2:14:1 phase grains, phase 2—Nd-rich phase, phase 3—oxide phases; (**b**) phase 4—a phase based on Fe–Nb; (scanning electron microscopy; marked phases correspond to those in Table 2).

In accordance with the microprobe analysis data shown in Table 3, the microstructure of a magnet prepared from the powder mixture with 2 wt.% Dy_3_Co_0.6_Cu_0.4_H*_x_* (Regime 1) was characterized by the presence of four structural components differing in the chemical composition, see Figure 5 (the phases are indicated by red numbers). 

The chemical composition of matrix grains (Phase 1 in Figure 5a) was close to the stoichiometric (Nd,R)_2_Fe_14_B composition. The presence of Dy in the matrix alloy did not allow us to unambiguously conclude the formation of the core–shell structure, but the presence of cobalt in 2:14:1 phase grains demonstrates the possibility of micro-alloying through the use of hydrogenated low-melting Co-containing compounds (the melting temperature was lower than the sintering temperature of Nd–Fe–B magnets). The Nd-rich phase (Phase 2 in Figure 5a) was characterized by a variable composition. Phase 3 (Figure 5a) corresponded to the oxide phases. In accordance with the literature data [19,20], they may be based on NdO, Nd_2_O_3_, or NdO_2_. The presence of a phase based on Fe–Nb in triple junctions (TJ) was observed (Phase 4, Figure 5b). This fact may be related to impurities in the industrially prepared alloy matrix.

**Figure 6:**
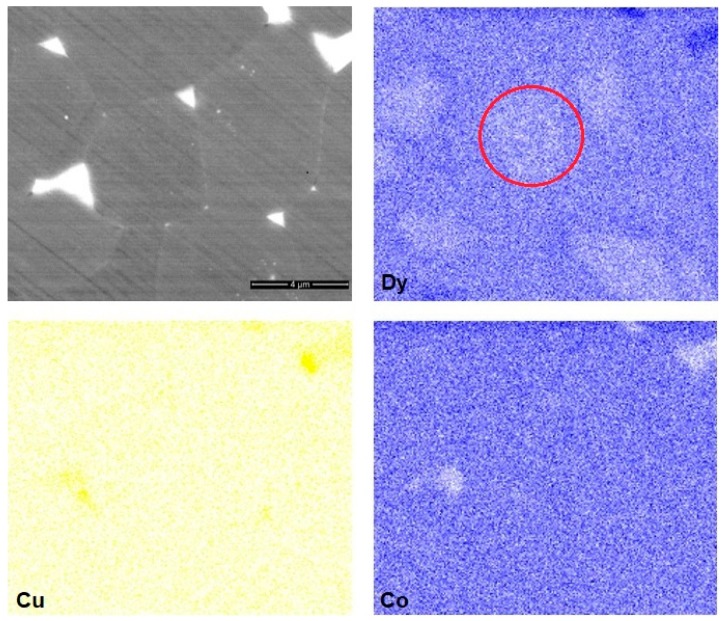
Co, Cu, and Dy mapping in 2:14:1 phase grains and triple junction phases of the Nd–Fe–B sintered magnet prepared from the powder mixture with 2 wt.% Dy_3_(Co,Cu). The red circle indicates the depletion of 2:14:1 phase grain in Dy (i.e. the formation of core-shell structure).

The distribution of rare earth elements, Co and Cu in the matrix grains, and in the intergranular Nd-rich phases (phase 2 in Figure 5a) in the sintered magnets prepared from the powder mixture with 2 wt.% of Dy_3_Co_0.6_Cu_0.4_H*_x_* addition was also investigated by X-ray mapping (see Figure 6). The nonuniform Dy distribution within the 2:14:1 phase grains could be observed. The depletion of triple junctions of Co and their enrichment in Cu should be noted in the case of the addition of Dy_3_Co_0.6_Cu_0.4_H*_x_*. The presence of reactive Dy powder (originating from DyH_2_ that was decomposed during sintering) ensures the diffusion of Dy atoms to the 2:14:1 phase lattice, since the atomic radius of Dy atoms is lower than that of Nd atoms. This led to ousting Nd atoms to peripheral areas. The diffusion coefficient of Nd atoms is lower than that of Dy atoms [21]; thus, the diffusion of Dy is more significant. Such an inequality of diffusion flows of atoms caused lattice stresses and resulted in the inhomogeneous Dy and Nd(Pr) distribution over the 2:14:1 phase grains. The core–shell structure (Dy-enriched shell and Dy-depleted core) is evident in Figure 6. 

**Figure 7:**
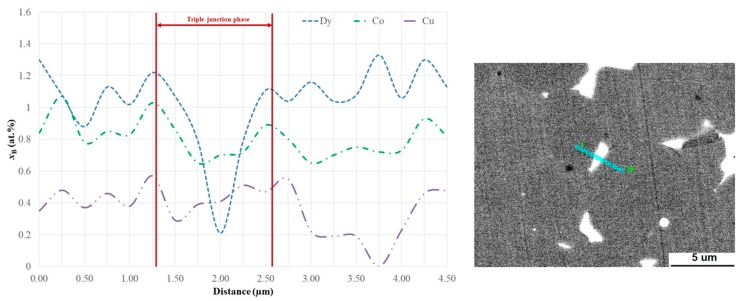
(**left**) Line chemical analysis over the triple junction phase and (**right**) corresponding SEM image with the analysis direction marked.

The other components of the Dy_3_Co_0.6_Cu_0.4_H*_x_* composition (i.e., Cu and Co) are also useful additions for Nd–Fe–B-based magnets. It is evident from Figure 6 and Figure 7 that Co evinced the tendency to incorporate the 2:14:1 phase grains, while the Cu enriched triple junction phases. The role of Cu in the grain-boundary restructuring and positive effects of Co on the coercivities of Nd–Fe–B magnets were reported in our previous work [22] and were also considered in [23,24,25,26,27,28,29,30,31]. 

### 3.4. Dependence of the Coercive Force (_j_H_c_) on the Heat Treatment Temperature

**Figure 8:**
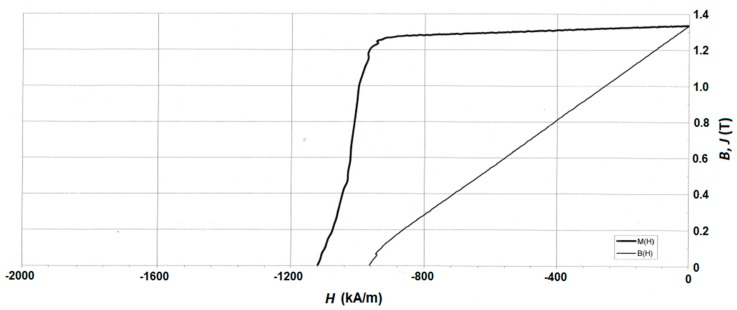
Magnetization reversal portions of hysteresis loop for the Nd–Fe–B sintered magnets prepared from the powder mixture with 2 wt.% Dy_3_Co_0.6_Cu_0.4_H_x_.

The magnetic properties (_j_*H*_c_) of the magnets (see Table 4 and Figure 8) prepared with the hydrogenated Dy_3_Co_0.6_Cu_0.4_ alloy were lower than those in the case of the application of the addition of the DyH_2_ [31]. One of the causes is the incomplete hydrogenation of the alloy (see Figure 3, XRD data) and, therefore, the incomplete occurrence of the grain boundary diffusion of the available Dy. The small quantity of the Dy_3_(Co,Cu) phase present in the Dy_3_Co_0.6_Cu_0.4_ alloy was subjected to hydrogenation. However, the value of *B*_r_ in the case of Dy_3_Co_0.6_Cu_0.4_H*_x_* was higher than that in the case of DyH_2_, which may be due to a difference in the Dy content in the chemical composition of the 2:14:1 phase. The difference in the rare-earth metal and Cu contents in the Nd-rich phases provided a lower value of *H*_k_ in the case of magnets with 2 wt.% Dy_3_Co_0.6_Cu_0.4_H_x_. The hysteretic properties of the Nd–Fe–B magnet, without the addition of hydride after optimal HT, are also shown in Table 4 for comparison. 

We assumed that the optimal HT for magnets of this type was in the range of 475 to 500 °C, as in the case of the magnets considered in [32,33,34]. Subsequent HT in this temperature range, which is performed after the optimal heat treatment (500 °C), will lead to an increase in the coercive force of magnets with 2 wt.% Dy_3_Co_0.6_Cu_0.4_H_x_. 

**Figure 9:**
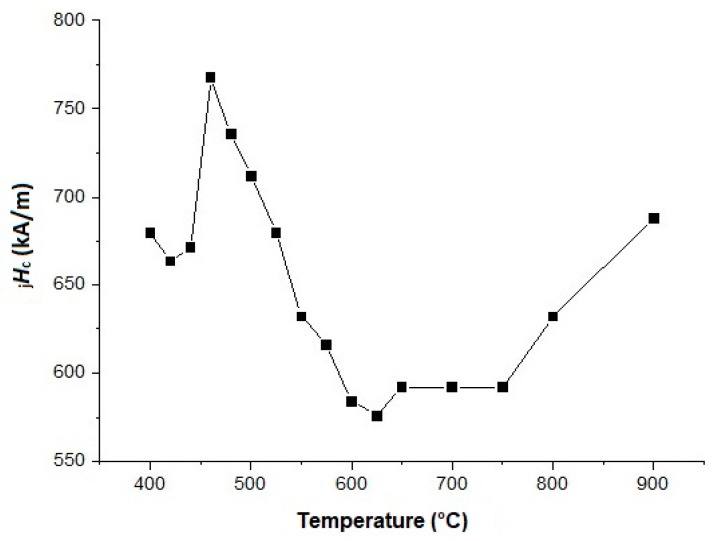
The dependence of _j_*H*_c_ on the heat treatment temperature of Nd–Fe–B-based magnet prepared from the powder mixture with 2 wt.% of Dy_3_Co_0.6_Cu_0.4_H_x_.

Figure 9 shows the variations of the coercive force (_j_*H*_c_) with changing heat treatment (HT) temperature. As can be seen from the data, after low-temperature HT in a range of 475–500 °C, _j_*H*_c_ demonstrated an abrupt increase. 

## 4. Conclusions

The phase composition of the Dy_3_Co_0.6_Cu_0.4_ alloy in the initial homogenized and hydrogenated states was studied. The alloy in the homogenized state was multiphase and contained the Dy_3_(Co,Cu) and Dy(Cu,Co) phases. During the hydrogenation of the alloy, the disproportionation or hydrogenolysis process took place, which, regardless of the multiphase composition of the initial alloy, resulted in the formation of DyH_2-3_ hydride and a fine (Co + Cu) mixture with small trace quantities of Dy_3_(Co,Cu) and Dy(Cu,Co). 

The study of the sintered Nd(RE)–Fe–B magnet prepared from the strip-cast alloy showed that Dy introduction via the two-component blending method (the hydogenated Dy_3_Co_0.6_Cu_0.4_ compound was added to the powder mixture) resulted in the formation of the core–shell structure of 2–14–1 phase grains. The efficient enhancement of the coercivity of Nd(RE)–Fe–B magnets, with a slight sacrifice of remanence, was demonstrated.

The positive effect of REM-alloy hydrogenated additions to the Nd–Fe–B powder mixture allows the possibility of introducing various components to the permanent magnets (heavy REMs, elements structuring grain boundaries, and restricting the magnet grain growth) at the preparation stage, rather than at the alloy-melting stage. This gives the possibility of using a unified initial alloy for the manufacture of magnets with improved (high-coercive or high-performance) magnetic characteristics.

## Figures and Tables

**Table 1:** The crystal structure type and lattice parameters of the phases in the Dy_3_(Co_0.6_Cu_0.4_) alloy.

Compound	Space Group	C	*a* (nm)	*b* (nm)	*c* (nm)	References
Dy_3_Co	Pnma	Fe_3_C	0.69650	0.93410	0.62330	[14]
Dy_3_(Co_1−x_Cu_x_)	Pnma	Fe_3_C	0.69331	0.93847	0.62564	This work
DyCu	Pm3$\bar{m}$	CsCl	0.34610	0.34610	0.34610	[15]
Dy(Cu_1−y_Co_y_)	Pm3$\bar{m}$	CsCl	0.34522	0.34522	0.34522	This work

**Table 2:** The chemical composition (at.%) of phases found in the microstructure of the Dy_3_Co_0.6_Cu_0.4_ alloy (the average value from three analysis).

Element/phase	Dy	Co	Cu
**Point 1**—Dy_3_(Co_0.6_Cu_0.4_)	75.5	15.1	9.5
**Point 2**—Dy(Co_1−y_Cu_y_)+Dy_3_(Co_0.6_Cu_0.4_)	68.1	9.0	22.9

**Table 3:** Chemical composition of phases observed in the structure of the Nd–Fe–B sintered magnet prepared from a powder mixture with 2 wt.% of Dy_3_Co_0.6_Cu_0.4_H_x_ (the values averaged for three measurements are presented).

Element/Phase	O	Dy	Al	Nb	Pr	Nd	Fe	Co	Cu
	(at.%)								
**Phase_1**		**1.0**	**0.6**	**0.2**	**3.1**	**10.3**	**83.0**	1.2	0.5
**Phase_2**		2.8	0.7	1.4	16.9	44.2	28.1	2.3	3.5
**Phase_3_1**	47.7	0.9	0.2	0.2	7.4	22.3	20.2	0.5	0.6
**Phase_3_2**	67.0	1.3	0.0	0.1	7.2	20.9	2.9	0.3	0.4
**Phase_3_3**	64.9	1.4	0.0	0.2	7.7	21.8	3.7	0.3	0.2
**Phase_4**		0.4	0.2	47.7	0.6	1.8	48.9	0.3	0.3

**Table 4:** Hysteretic properties of sintered magnets prepared from the powder mixtures with 2 wt.% Dy_3_Co_0.6_Cu_0.4_H*_x_* and DyH_2_ and optimally heat treated at 500 °C for 2 h; *B*_r_ = remanence of magnetic flux density; _j_*H*_c_ = coercivity of magnetic polarization; *H*_k_ = parameter adopted as a criterion of coercivity (i.e., the magnetic field determined at 0.9 × *B*_r_); (*BH*)_max_ = maximum energy product; HT = heat treatment.

Addition/Annealing Conditions	*B*_r_	_j_ *H*_c_	*H*_k_	*(BH)*_max_
	(T)	(kA/m)	(kA/m)	(kJ/m^3^)
Dy_3_Co_0.6_Cu_0.4_H_x_/optimal HT	1.34	1120	968	336
DyH_2_/optimal HT	1.29	1309	1262	322
0 wt.% of addition/optimal HT ^∗^ [9]	1.36	1000	850	358

^∗^ The initial Nd–Fe–B alloy contains 0.5 wt.% Dy.
